# Apoptotic Pathways in Pemphigus

**DOI:** 10.1155/2010/456841

**Published:** 2010-06-15

**Authors:** Meryem Bektas, Puneet Jolly, David S. Rubenstein

**Affiliations:** ^1^Department of Dermatology, University of North Carolina-Chapel Hill School of Medicine, Chapel Hill, NC 27599, USA; ^2^Department of Pharmacology, University of North Carolina-Chapel Hill School of Medicine, Chapel Hill, NC 27599, USA; ^3^Lineberger Comprehensive Cancer Center, University of North Carolina-Chapel Hill School of Medicine, Chapel Hill, NC 27599, USA

## Abstract

Pemphigus is a group of human autoimmune blistering diseases of the skin in which autoantibodies to desmosome cadherins induce loss of cell-cell adhesion (acantholysis). In addition to steric hindrance and activation of intracellular signaling, apoptosis has been suggested to contribute to the mechanism by which pathogenic IgG induces acantholysis. We review the current literature examining the role of apoptosis in pemphigus. Current data suggest that apoptosis is not required for blister induction, but that activation of proapoptotic proteins, including caspase cysteine proteinases, may sensitize cells to the acantholytic effects of pemphigus IgG.

## 1. Introduction

Pemphigus is a group of autoimmune blistering diseases characterized by loss of keratinocyte cell adhesion that leads to clinical blister formation. Pemphigus vulgaris (PV) and pemphigus foliaceus (PF) are the two main variants. In these disorders, autoantibodies to desmosome cadherins induce blister formation. In PF, pathogenic IgG target the desmosome cadherin desmoglein-1 (dsg-1) [1], inducing loss of adhesion in the subcorneal region of epidermal epithelia; whereas, in PV, antibodies to desmoglein-3 (dsg-3) in mucosal PV [2] and to dsg-3 and dsg-1 in mucocutaneous PV [3–5] induce loss of adhesion in the suprabasal layer.

## 2. Signal Transduction and Acantholysis

When injected into neonatal mice, IgG fractions from pemphigus patients induce blister formation reproducing the histology, immunohistology, and clinical appearance of the human disease [6, 7]. This seminal observation provided the evidence that the autoantibodies themselves were pathogenic, inducing blister formation in the target organ, the skin. A variety of mechanisms have been proposed to explain how pemphigus autoantibodies disrupt keratinocyte cell-cell adhesion. The observation that pemphigus IgG were directed against the desmosomal cadherins dsg-1 and dsg-3 led to the suggestion that the IgG inhibited trans interactions of desmosome adhesion proteins across the adhesive interface, a mechanism know as the steric hindrance hypothesis. Alternative hypotheses have been suggested, including antibody induction of intracellular signaling that leads to loss of adhesion, and antibody induction of keratinocyte apoptosis. 

Changes in cell-cell adhesion are often accompanied by significant alterations in the biology of the cell. When cells become adherent to adjacent cells, they often cease to migrate and proliferate, a concept referred to as contact inhibition and loss of which often characterizes the malignant phenotype of cancer cells. In contrast, when cells lose adhesion to their neighbors, they can undergo apoptosis, or proliferate, and, or migrate. Which of these later fates occur is often context dependent. For example, loss of adhesion to and migration from the wound margin is typical of the biology of a keratinocyte migrating into a healing wound. Therefore, it is not surprising that alterations in cell adhesion complexes initiate, inhibit, and/or regulate signaling processes. 

Kitajima's group was the first to describe the activation of signaling pathways in cells exposed to pemphigus IgG. They first reported increased intracellular calcium levels in keratinocytes treated with sera from PV patients [8]. In subsequent studies, using either PV sera or fractions enriched for pathogenic IgG, they demonstrated activation of additional signaling molecules including protein kinase C [9] and phospholipase C [10]. Other signaling events have been observed in pemphigus treated keratinocytes and may contribute to loss of or to biologic transitions induced by altered adhesion [11–18].

## 3. Apoptosis

Programmed cell death is a mechanism used by multicellular organisms to remove unwanted cells. For example, the shaping of tissues during development is a coordinated process of proliferation, migration, and cell death. In fully developed organisms, tissues that continuously proliferate, including the skin, must balance cell proliferation and death to prevent unchecked growth. In pathologic states, including actinic damage that may lead to neoplastic transformation, apoptosis is employed to remove from the host tissue these potentially damaging cells. Apoptosis is characterized by a number of well recognized cytological and biochemical features including, (i) nuclear condensation, (ii) activation of caspase cysteine proteinases, (iii) DNA fragmentation at regular intervals resulting in DNA laddering, (iv) cell shrinkage, and (v) membrane blebbing (reviewed in [19]).

 A number of reports indicate that apoptosis occurs as a consequence of exposure of human keratinocytes to pemphigus sera or purified IgG fractions, observed in both in vitro tissue culture systems, in vivo utilizing passive transfer mouse models, or in human patient skin biopsies. Induction of apoptosis or of proapoptotic proteins by pemphigus IgG may be (i) part of the mechanism by which sera and IgG induce acantholysis or (ii) a consequence of loss of adhesion and a result of acantholysis. Apoptosis has been proposed to have a role in the mechanism by which pemphigus IgG induce acantholysis [20, 21]. Initial reports examined histological sections of skin biopsies and TUNEL positive keratinocytes, indicative of DNA fragmentation characteristic of cells undergoing apoptosis, in both lesional PF [22] and PV [20] and in perilesional pemphigus skin [23]. Markers for apoptotic cells, apart from TUNEL include components of the proapoptotic pathway such as caspases, PARP, or Fas. These proapoptotic markers have also been used to investigate the presence of apoptotic cells in PV patient skin biopsies.

## 4. Immune Cells and Fas/FasL

Although there is a fairly broad consensus that apoptosis can be observed in pemphigus IgG induced disease processes, there remains an ongoing discussion about whether apoptosis is a (i) cause, (ii) result, or (iii) “byproduct” of acantholysis. For example, modeling anoikis, it would be straightforward to think of apoptosis as consequence of acantholysis. Anoikis is a special form of apoptosis of epithelial cells triggered when cells become detached from the substratum. Disruption of integrin-extracellular matrix binding is a main trigger for anoikis [24]. In contrast, the main adhesive forces between cells in the suprabasal layers of the epidermis are adherens junctions and desmosomes; therefore, anoikis seems to be unlikely to be important in pemphigus. Furthermore, it has been reported that PV IgG, but not normal human IgG, cause cell death as measured by trypan blue exclusion in suspension keratinocytes [25]. The ability of pemphigus IgG to induce cell death in nonadherent keratinocytes would seemingly exclude the loss of cell attachment as a trigger for pemphigus IgG induced cell death. 

As early as 1994, Sayama and coworkers, and later other groups, reported upregulation of soluble Fas ligand in either PV patient sera and, or skin, suggesting that apoptosis in the target organ skin is triggered via the Fas/FasL system [20, 23, 26, 27]. Importantly, anti-FasL blocking antibodies only partially inhibited this proapoptotic effect of pemphigus sera on cultured keratinocytes suggesting that other mechanisms may contribute to induction of apoptosis. Rodrigues and colleagues also reported observing TUNEL positive keratinocytes in perilesional biopsies in patients with Fogo selvagem, the endemic form of PF [28]. Interestingly, these authors also noted the presence of the inflammatory cytokines TNF-*α*, IFN-*γ*, and IL-1 in the lesion exudate which led them to suggest that inflammatory mediators could contribute to the induction of apoptosis in pemphigus. Similarly, Pacheco-Tovar and colleagues suggested that CD8+FasL+ lymphocytes in pemphigus skin may have a role in inducing apoptosis [26]. In contrast, other groups have suggested that pemphigus associated apoptosis is independent of changes in Fas-ligand levels. For example, Reich and colleagues did not see increased levels of FasL in PNP patient sera, despite observing increased apoptotic cells in PNP skin biopsies [29]. 

Genetic polymorphisms in genes encoding apoptotic factors have been identified in autoimmune disorders. Köhler and Petzl-Erler reasoned that if apoptosis has a role in pemphigus, then genetic polymorphisms in apoptotic factors might similarly explain the susceptibility of certain individuals to this disease; however, they were unable to find an association between specific polymorphisms in the genes encoding either the p53 or BAX in a Brazilian focus of endemic PF [30].

 Taken together, pemphigus lesions have been described with or without the infiltration of immune cells suggesting that inflammation is not necessarily an early event nor required for pemphigus disease induction. Nevertheless, the latter observation could explain the contradictory finding of the presence or absence of FasL in pemphigus lesions by different labs.

## 5. The Temporal Association of Acantholysis and Apoptosis

Apoptosis leads to cell death; whereas, in pemphigus, the engagement of cell surface antigens by pathogenic leads to loss of adhesion, a point discussed by Grando and colleagues in a recent commentary [31]. The timing of these events and their relation to one another, that is the loss of adhesion and activation of proapoptotic proteins, has been subject to debate and investigation. Grando uses the term apoptolysis to suggest a relationship between the activation of apoptotic proteins including caspases, and acantholysis emphasizing that caspase activation and acantholysis can proceed in the absence of cell death. Namely, that caspases could serve cell regulatory functions in addition to apoptosis. In support of this hypothesis is data demonstrating that many desmosome components as well as intermediate filaments can serve as caspase substrates [32–35]. Thus, caspases may have a role in regulating desmosome protein turnover under normal as well as pathophysiologic states. 

Differing reports on the role of apoptosis in pemphigus IgG mediated acantholysis may reflect the different systems and experimental conditions used. For example, different keratinocyte culture systems, including primary human keratinocytes, squamous cell carcinoma cell lines, and the immortalized HaCat keratinocyte cell line, have been used to examine in vitro the role of apoptosis in pemphigus acantholysis. Similarly, cell cultures that have been passaged multiple times, believed to reproduce some of the biochemical and physiologic alterations associated with aging, appear more susceptible to the apoptosis inducing activity of pemphigus IgG [36]. Utilizing cell culture systems, extended incubations are often used to examine the ability of pemphigus sera or pemphigus IgG to induce keratinocyte apoptosis. Incubations with pemphigus sera and/or IgG from 8 to 72 hours have been used in experiments in which activation of proapoptotic markers including TUNEL positivity have been observed at typically later time points [37]. Moreover, monolayer cultures cannot fully replicate the in vivo situation, as cells in culture are proliferating; whereas, the cells affected and involved in pemphigus disease processes are suprabasal keratinocytes that have ceased to proliferate, but are differentiating. Differences in proliferation and differentiation could lead to altered susceptibility to apoptotic stimuli. Skin biopsies are a snapshot in time of the disease progression and reported differences in the biopsies may be attributed to different stages of disease progression. While very early disease lesions may be negative for apoptotic markers, late lesions may show apoptosis as well as immune cell infiltrates.

## 6. The In Vivo Effect of Caspase Inhibitors

Li and colleagues explored the ability of caspase inhibitors to block acantholysis in vivo using the PF passive transfer mouse model [38]. They reported that pretreatment of neonatal mice with 0.034–6.8 *μ*g/g body weight of the caspase inhibitors Ac-DEVD-cmk or Boc-D-fmk prevented PF IgG induced blister formation in the mice at 20 hours. Interestingly, in time course studies they observed increased amounts of the proapoptotic factor Bax initially and subsequent decreases in the antiapoptotic factor Bcl-xL at later time points, an observation they suggest supports a role for induction of apoptosis via the mitochondrial pathway. They interpret these results to implicate caspase activity in the mechanism of acantholysis. This report stands in contrast to an in vitro study by Schmidt and colleagues suggesting that caspase inhibitors do not block acantholysis [39]. Using the immortalized HaCat keratinocyte cell line as well as normal human epidermal keratinocyte cultures as in vitro model systems, they observed PV IgG induced keratinocyte dissociation and cytokeratin retraction without observable markers of apoptosis such as nuclear condensation, TUNEL staining, and caspase-3 activation. The caspase inhibitor z-VAD-fmk did not block PV IgG mediated acantholysis in cultured keratinocytes. In addition to using pharmacologic inhibitors of apoptosis, genetic approaches also failed to demonstrate a role for apoptosis in PV IgG mediated acantholysis. In these experiments, overexpression in HaCat cells of the Fas-associated death domain-like interleukin-1-*β*-converting (FLICE)-like inhibitory proteins FLIP_L_ and FLIP_S_, inhibitors of caspase-8 mediated activation and apoptosis, blocked Fas induced apoptosis, but failed to block PV IgG mediated acantholysis. An additional important finding in the above mentioned report is that not all lesions from the same patients, particularly very early ones, showed markers of apoptosis, an observation suggesting that apoptosis is not essential for acantholysis.

## 7. Pemphigus, P38MAPK, Acantholysis, and Apoptosis

In a series of experiments initially designed to look at signaling pathways downstream of changes in desmosome mediated cell-cell adhesion, our research group identified activation of p38 mitogen activated protein kinase (MAPK) as a necessary event for pemphigus IgG induced acantholysis [40]. Phosphorylation is a common, reversible modification that regulates protein structure, function, and activity. Because changes in protein phosphorylation are common in signal transduction cascades and can be readily detected and quantified with radioactive phosphate, we designed a strategy to screen for signaling downstream of pemphigus IgG induced changes in desmosome mediated adhesion. Primary human keratinocytes were loaded with ^32^P-H_3_PO_4_ and then exposed to purified PV IgG for 30 minutes. Protein extracts were prepared from labeled cells and separated by two-dimensional gel electrophoresis. Radioactive proteins were detected by autoradiography and quantified by phosphoimage analysis. Using this approach, we identified several radioactive spots whose signal was consistently increased in samples treated with PV IgG, but not in normal human IgG nor buffer treated controls. Through this screen we first identified the small heat shock protein (HSP) 27 and p38MAPK as components of a putative signaling cascade downstream of PV IgG induced changes in desmosome adhesion. Because p38MAPK was known to be upstream of HSP27, it was not surprising that keratinocytes pretreated with the p38MAPK inhibitors SB202190 or SB203580, but not the inactive analog SB202474, failed to induce HSP27 phosphorylation when treated with PV IgG. What was surprising was that the p38MAPK inhibitors also blocked PV IgG induced actin reorganization and keratin intermediate filament retraction. This observation suggested that activation of p38MAPK might not be a signal activated by loss of adhesion, but could in fact be part of the mechanism by which PV IgG induced loss of adhesion. These observations were later confirmed using passive transfer mouse models of both PV [41] and PF [42]. Phosphorylation of both HSP27 and p38MAPK were observed in skin biopsies from PV and PF IgG treated neonatal mice. Significantly, in mice pretreated with p38MAPK inhibitors, p38MAPK and HSP27 phosphorylation and blister formation were blocked. Activation of p38MAPK in keratinocytes treated with PV IgG has now been independently confirmed by several labs actively investigating the mechanism of acantholysis [12, 16, 43]. 

Our earlier studies had demonstrated a role for p38MAPK in the mechanism by which pemphigus IgG induce acantholysis. Based on (i) the known roles of p38MAPK and HSP27 in regulating both the actin and intermediate filament cytoskeletons [44–50] and (ii) the ability of p38MAPK inhibitors to block PV IgG induced actin reorganization and keratin intermediate filament collapse in keratinocyte tissue cultures [40], we had proposed that p38MAPK was acting upstream of and regulating both actin and keratin intermediate filament cytoskeletal changes induced by pemphigus IgG. Recent work in our lab has also demonstrated a role for p38MAPK in regulating pemphigus IgG induced desmoglein endocytosis [51]. 

Because of the well-characterized involvement of p38MAPK in apoptosis [52–54], we initiated a series of experiments to investigate the potential relationship of PV IgG induced activation of p38MAPK to apoptosis [55]. In this series of experiments, both tissue culture and animal model systems were employed. Primary human keratinocyte cultures allowed us to very accurately follow the time course of biochemical changes induced by pemphigus IgG; whereas, utilization of the pemphigus passive transfer mouse model allowed us to determine if the events observed in vitro were reflective of the in vivo state. Primary human keratinocytes were exposed to PV IgG and examined biochemically and by confocal immunofluorescent microscopy at various times after addition of PV IgG to the cultures. Interestingly, two sequential peaks of p38MAPK activation were observed, the first beginning within minutes after addition of PV IgG, peaking at 30 minutes, and then dropping down to baseline by 4 hours. A second extended peak of p38MAPK activation was observed to begin at 6 hours and continued out to 10 hours. Analogous to the observations in tissue culture, a similar biphasic activation of p38MAPK in the skin was observed in the passive transfer PF mouse model. An initial peak of p38MAPK activation was seen at 2 to 4 hours after subcutaneous administration of purified PF IgG to neonatal C57BL/6J mice which dropped down to baseline levels at 6 hours. A second peak of p38MAPK was observed in murine skin at 8 hours post PF IgG injection and was sustained out to at least 21 hours post injection. Along with p38MAPK activity, markers of apoptosis, including TUNEL staining, PARP cleavage, and caspase-3 cleavage were also examined at sequential time points after treatment with pemphigus IgG. In both primary human keratinocyte cultures and the passive transfer mouse model, markers of apoptosis were late events occurring coincident or subsequent to the second peak of p38MAPK activation. In the passive transfer mouse model, acantholysis was readily apparent at 21 hours after treatment with PF IgG; however, neither cleaved PARP nor cleaved caspase-3 could be detected at either the 21- or 24-hour-time points, well after the second peak of p38MAPK activity had begun. It was not until 30 hours post PF IgG injection that these markers of apoptosis (cleaved PARP and cleaved caspase-3) were detected by immunoblot of skin extracts. Similarly, only in skin biopsies from mice treated with PF IgG for times exceeding 21 hours were increases in TUNEL positive keratinocytes detected. 

**Figure 1:**
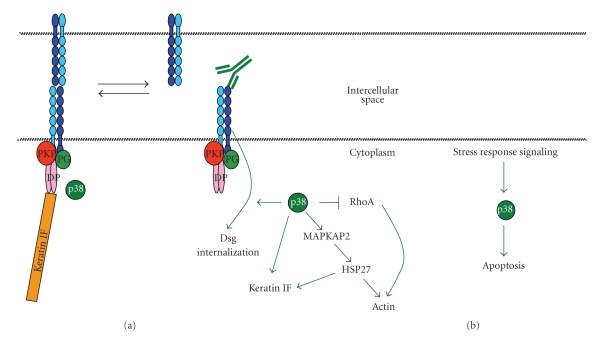
Pemphigus, p38MAPK, acantholysis and apoptosis. Two sequential peaks of p38MAPK activation are observed when keratinocytes are exposed to either PV or PF IgG. (a) Pemphigus IgG binds to dsg and biases the equilibrium of desmosome assembly/disassembly towards disassembly which is linked by an, as yet, undefined mechanism towards activation of p38MAPK. Subsequent p38 dependent alterations in the cell state include RhoA inactivation, dsg endocytosis, HSP27 phosphorylation, keratin intermediate filament retraction, actin, and loss of cell-cell adhesion (acantholysis). (b) A second late peak of p38 activity is observed that is likely a stress response signal induced by loss of cell-cell adhesion and leads to activation of proapoptotic pathways including caspase-3 activation.

To investigate the relationship of the first and second peak of p38MAPK activity to acantholysis, and apoptosis, inhibitor experiments were performed in both primary human keratinocyte culture and passive transfer mouse models. By either pretreating cultures or mice with p38MAPK inhibitors prior to pemphigus IgG or at a time after the first, but prior to the second peak of p38MAPK activity, either both peaks or the second peak of p38MAPK activity could be selectively inhibited. Utilizing this approach, inhibition of the first, but not second, peak of p38MAPK activity inhibited blister formation in vivo and cytokeratin retraction in vitro [55]. We interpreted these results to indicate that the first, but not second peak, of p38MAPK activity observed after exposure to pemphigus IgG was part of the mechanism of acantholysis. Importantly, although it failed to inhibit cytokeratin retraction and acantholysis, selective inhibition of the later second peak of p38MAPK activity inhibited caspase-3 activation in vivo. Thus, the time course studies revealed that apoptosis occurs at or after the second peak of p38MAPK activation and that inhibition of this later peak of p38MAPK activity blocked activation of the proapoptotic proteinase caspase-3, but not acantholysis. Collectively, these observations suggest that the earlier peak of p38MAPK activation is part of the mechanism leading to acantholysis; whereas, the later peak of p38MAPK and apoptosis are subsequent to and likely not essential for acantholysis (Figure 1).

 Interestingly, pemphigus IgG induced cytokeratin retraction may trigger apoptosis as suggested by a recent report from the Omary lab implicating a role for keratin intermediate filaments in the induction of apoptotic pathways. Mutations in the hepatocyte keratin K8 were associated with altered mitochondrial morphology and increased susceptibility to proapoptotic stimuli [56]. Null K8 or K8 mutants show decreased mitochondrial size, a shift from diffuse to cortical distribution and clumping, and increased mitochondrial release of cytochrome c from mutants. Therefore, it may be the collapse of the intermediate filament network in pemphigus that contributes to activation of apoptosis in these cells.

## 8. Making Sense of It All

From studies to date, it is clear that activation of proapoptotic proteins, including caspases, occurs in pemphigus. Although studies from our lab and from Waschke's group indicate that apoptosis is not essential for acantholysis to proceed, Li and coworkers have shown that caspase inhibitors can block acantholysis in the PF passive transfer mouse model. The challenge of resolving these seemingly mechanistically divergent observations is not as difficult as it would first appear. 

Although caspases are cysteine proteinases implicated in apoptosis, they may have additional nonapoptotic biologic functions [57] including regulating desmosome assembly/disassembly. This is particularly relevant because data is accumulating that desmosomes are dynamic complexes in which the adhesive structure is maintained by the equilibrium between desmosome assembly and disassembly. In pemphigus, pathogenic antidsg antibodies promote dsg internalization [14, 15, 51, 58–61] and bias the equilibrium towards disassembly. Similarly, caspases have been shown to cleave desmosome proteins and therefore may be important to the physiologic cycling/turnover of dsg in keratinocytes. The desmosome proteins dsg-3, dsg-1, plakoglobin, and desmoplakin [32, 33], as well as intermediate filaments [34, 35], have all been shown to undergo caspase-dependent cleavage. Furthermore, caspases may also regulate matrix metalloproteinase (MMP) dependent cleavage of desmogleins [33]. In A431 epithelial cells induced to undergo apoptosis by UV exposure, UV-induced MMP cleavage of dsg-1 ectodomain could be inhibited by both MMP specific inhibitors as well as by the caspase inhibitor ZVAD-fmk [33]. Staphylococcal Scalded Skin Syndrome (SSSS) demonstrates that proteolytic cleavage of desmogleins can cause acantholysis. In SSSS, the Staphylococci secrete exfoliative toxin, a serine proteinase that cleaves the ectodomain of dsg-1; thereby, disrupting desmosome mediated adhesion in the subcorneal layers of epidermal epithelia and yielding a phenotype mimicking the subcorneal blisters of PF [62, 63]. Thus, caspase-dependent proteolysis has the potential to augment the acantholytic effects of pemphigus IgG. Analogously, blocking caspase dependent proteolysis of desmosome proteins and intermediate filaments may stabilize keratinocyte cell-cell adhesion thereby increasing their resistance to pemphigus IgG induced acantholysis.

 Pemphigus induced acantholysis is a disease specific model system that has facilitated the investigation of desmosome dynamics, adhesion, and the mechanisms by which alterations in desmosome structure impact intracellular signaling and regulatory pathways. Although activation of proapoptotic pathways appears to be a late event and may not be essential for blistering in pemphigus, activation of components of apoptotic signaling, including caspase family member proteinases, could augment the blistering response as downstream effects of p38MAPK activation. Adhesion is a dynamic process that is linked to other biologic events including cell migration, proliferation, differentiation, and death. Changes in desmosome adhesion impact these processes and is likely to involve multiple components and signaling pathways. Elucidating these components and pathways will provide fertile ground for future investigations.

## Abbreviations

dsg-1: Desmoglein 1
dsg-3: Desmoglein 3
HSP: Heat shock protein
MMP: Matrix metallo-proteinase
p38MAPK: p38 mitogen activated protein kinase
PF: Pemphigus foliaceus
PNP: Paraneoplastic pemphigus
PV: Pemphigus vulgaris
SSSS: Staphylococcal Scalded Skin Syndrome.
